# Marginal Ascorbate Status (Hypovitaminosis C) Results in an Attenuated Response to Vitamin C Supplementation

**DOI:** 10.3390/nu8060341

**Published:** 2016-06-03

**Authors:** Anitra C. Carr, Juliet M. Pullar, Stephanie M. Bozonet, Margreet C. M. Vissers

**Affiliations:** Department of Pathology, University of Otago, Christchurch, P.O. Box 4345, Christchurch 8140, New Zealand; juliet.pullar@otago.ac.nz (J.M.P.); stephanie.bozonet@otago.ac.nz (S.M.B.); margreet.vissers@otago.ac.nz (M.C.M.V.)

**Keywords:** hypovitaminosis C, vitamin C supplementation, human intervention study, recommended dietary intake, body weight

## Abstract

Inadequate dietary intake of vitamin C results in hypovitaminosis C, defined as a plasma ascorbate concentration ≤23 μmol/L. Our objective was to carry out a retrospective analysis of two vitamin C supplementation studies to determine whether supplementation with 50 mg/day vitamin C is sufficient to restore adequate ascorbate status (≥50 μmol/L) in individuals with hypovitaminosis C. Plasma ascorbate data from 70 young adult males, supplemented with 50 or 200 mg/day vitamin C for up to six weeks, was analyzed. Hypovitaminosis C status was identified based on plasma ascorbate being ≤23 μmol/L and the response of these individuals to vitamin C supplementation was examined. Of the participants consuming 50 mg/day vitamin C for up to six weeks, those with hypovitaminosis C at baseline achieved plasma concentrations of only ~30 μmol/L, whereas the remainder reached ~50 μmol/L. Participants who consumed 200 mg/day vitamin C typically reached saturating concentrations (>65 μmol/L) within one week, while those with hypovitaminosis C required two weeks to reach saturation. Regression modelling indicated that the participants’ initial ascorbate status and body weight explained ~30% of the variability in the final ascorbate concentration. Overall, our analysis revealed that supplementation with 50 mg/day vitamin C, which resulted in a total dietary vitamin C intake of 75 mg/day, was insufficient to achieve adequate plasma ascorbate concentrations in individuals with hypovitaminosis C. Furthermore, increased body weight had a negative impact on ascorbate status.

## 1. Introduction

Vitamin C (ascorbate) is an essential dietary micronutrient that, due to its role as a cofactor for a number of functionally-related metalloenzymes [1,2], has many important biosynthetic and regulatory functions in the body. Inadequate dietary intakes result in hypovitaminosis C (defined as a plasma concentration ≤23 μmol/L [3]) and the potentially fatal deficiency disease scurvy [4]. A relatively low intake of vitamin C (~10 mg/day) is sufficient to prevent the physical manifestations of scurvy and it is, therefore, rare in the Western world [5]. However, epidemiological studies have indicated that a significant proportion of these populations are affected by hypovitaminosis C [6,7,8]. Individuals with hypovitaminosis C can exhibit signs of sub-clinical vitamin C deficiency, such as fatigue and depression, and can be at risk of developing overt scurvy [9,10].

Historically, recommended dietary intakes (RDIs) for vitamin C have been based on the prevention of scurvy, with a margin of safety. Australia and New Zealand (collectively known as Australasia) and the United Kingdom have the lowest RDI for vitamin C in the developed world, with 40 to 45 mg/day being recommended. This is approximately half the intake recommended in North America and many European and Asian countries [11,12,13]. The disparity between the international RDIs for vitamin C is due to the different criteria used to determine them, *i.e.*, the prevention of deficiency *versus* potential health benefits.

Numerous epidemiological studies indicate that higher intakes of vitamin C are likely to have a role in the prevention of chronic diseases (reviewed in [14,15]). Therefore, we and others have recommended that the RDI for vitamin C be increased to 120 or even 200 mg/day to take into account its additional health benefits [14,15]. In the late 1990s the United States’ Food and Nutrition Board undertook a review of their Nutrient Reference Values for the antioxidant vitamins, including vitamin C [12]. The new criteria were based on an amount of vitamin C that provided antioxidant protection [14,16] and was derived from near maximal neutrophil concentrations with minimal urinary loss [9]. As a result, the North American RDI for vitamin C was increased from 60 mg/day to 90 mg/day for adult men and 75 mg/day for adult women [12]. Higher vitamin C intakes were recommended for pregnant and lactating women due to additional requirements for fetal and infant growth and nutrition, and for smokers due to faster metabolic turnover of the vitamin [12].

Recently the German, Austrian, and Swiss Nutrition Societies revised the reference values for the required intake of vitamin C [17]. Their criteria for the average vitamin C requirement in healthy adults is the amount of vitamin C that compensates for metabolic losses and ensures a fasting ascorbate plasma level of 50 μmol/L, in agreement with that considered ‘adequate’ by the European Food Safety Authority [13]. Their calculated average requirement for adult men is 91 mg/day, which gives a recommended intake of 110 mg/day. The requirement for women is extrapolated from these values, and is related to body weight, giving a recommended intake of 95 mg/day for adult women. Higher intakes were recommended for pregnant and lactating women and smokers due to their higher vitamin C requirements [17].

In contrast, the Australasian RDI for vitamin C of 45 mg/day for both men and women [11] is based on the Estimated Average Requirement (EAR) for vitamin C, which is 30 mg/day [11]. The EAR is the intake estimated to meet the nutritional requirements of 50% of adults, and is derived from an intake at which body content is halfway between tissue saturation and the point at which clinical signs of scurvy appear. Health outcomes are not used for assessing EAR criteria [11] and this may be due to repeated findings in large randomized placebo-controlled trials for vitamin C that have shown little or no effect of supplementation [18,19,20]. However, the ascorbate status of these cohorts was not assessed prior to intervention, with a subsequent failure to exclude participants who already had adequate to saturating ascorbate levels [3].

We recently carried out a number of vitamin C bioavailability studies in young adult men supplemented with vitamin C in the form of chewable tablets or from gold kiwifruit [21,22,23,24]. During sub-group analysis of our study cohorts we observed some individuals whose plasma ascorbate concentrations did not rise to adequate levels (*i.e*., ≥50 μmol/L [13]) following supplementation. Here we report on a retrospective data analysis of 70 individuals supplemented with 50 or 200 mg/day vitamin C which indicates that individuals with hypovitaminosis C have an attenuated response to supplementation with 50 mg/day vitamin C.

## 2. Materials and Methods

### 2.1. Participants and Study Design

This study comprised a retrospective analysis of data collected during two vitamin C intervention studies (2011–2014, *n* = 70 participants). All procedures involving human participants were approved by the Upper South Regional Ethics Committee (#URA/11/02/003) and the Health and Disability Ethics Committees (#13/STH/105/AM01). The studies were registered with the Australian New Zealand Clinical Trials Registry (ACTRN12611000162910 and ACTRN12613000989741).

Participants and study designs have been described in detail in previous publications [22,23,24]. Briefly, non-smoking males aged 18–35 years were recruited from local tertiary institutes and a fasting venous blood sample was drawn at screening to determine plasma ascorbate status. Plasma ascorbate was measured using HPLC with electrochemical detection as described previously [21]. Individuals with plasma ascorbate concentrations <50 μmol/L were recruited for the studies and all provided written informed consent to participate in these studies.

Participants received either 50 or 200 mg/day vitamin C in the form of orange-flavored chewable tablets comprising ascorbic acid and sodium ascorbate (provided by Tishcon Corp., Westbury, NY, USA) or Gold kiwifruit (*Actinidia chinensis*, provided by Zespri International Ltd, Mount Maunganui, New Zealand). We have previously shown that the bioavailability of synthetic and kiwifruit-derived vitamin C are equivalent [22,25] so the data from these two forms of supplementation were combined. Specifically, the low vitamin C group (*n* = 35) comprised 18 participants who received half a kiwifruit/day and 17 participants who received 50 mg/day vitamin C tablets [22]. The high vitamin C group (*n* = 35) comprised 30 participants who received two kiwifruit/day (*n* = 17 [23] and *n* = 13 [24]) and five participants who received 200 mg/day vitamin C tablets (as part of a previously unpublished crossover extension of [22]).

The participants underwent a lead-in phase of up to five weeks, an intervention phase of four to six weeks and a washout phase of four weeks. We have previously shown that four weeks is sufficient time for 50 mg/day supplementation to reach steady-state plasma levels [21,22]; less time is required for 200 mg/day supplementation [23]. During the lead-in phase, the participants were encouraged to control their dietary vitamin C intake by avoiding consumption of juices and other vitamin C-fortified beverages, and by substituting vitamin C-rich foods with low vitamin C-containing foods. This dietary regime was followed for the duration of the study.

Fasting venous blood samples were drawn weekly throughout the studies to monitor the participants’ plasma ascorbate concentrations derived from both their normal daily diet and the intervention. Participants from the first study (*n* = 57) also completed seven-day food and beverage records at the beginning of the study, pre- and post-intervention, and post-washout to monitor their dietary vitamin C intake. Analysis of the seven-day food and beverage records was carried out as described previously [21].

### 2.2. Statistical Analysis

Data were pooled from the two intervention studies [22,23,24] and individuals with hypovitaminosis C were identified based on a plasma ascorbate concentration of ≤23 μmol/L at study entrance. Data are represented as mean ± SD. The differences between paired and unpaired data were determined by two-tailed *t*-test and *p* values ≤ 0.05 were considered significant. Backward stepwise multiple linear regression analysis was conducted using SPSS software (version 22, IBM Corp, Armonk, NY, USA) to determine independent predictors of plasma vitamin C concentration.

## 3. Results

### 3.1. Group Characteristics at Study Entrance

The characteristics of the cohort at study entrance are shown in Table 1. For the current analysis, the two intervention groups (consuming 50 or 200 mg/day vitamin C) were subdivided into those with plasma ascorbate concentrations >23 μmol/L and those with ≤23 μmol/L at study entrance; this is considered the ‘marginally deficient’ or ‘hypovitaminosis C’ cut-off [10]. Although the ≤23 μmol/L sub-group within the 50 mg/day intervention group had a slightly higher BMI than the >23 μmol/L sub-group, there were no significant differences in BMI between the two ≤23 μmol/L sub-groups (Table 1). There were no other significant differences in anthropometric measures between the sub-groups or intake groups.

### 3.2. Plasma Ascorbate in the 50 mg/Day Intervention Group

**Figure 1:**
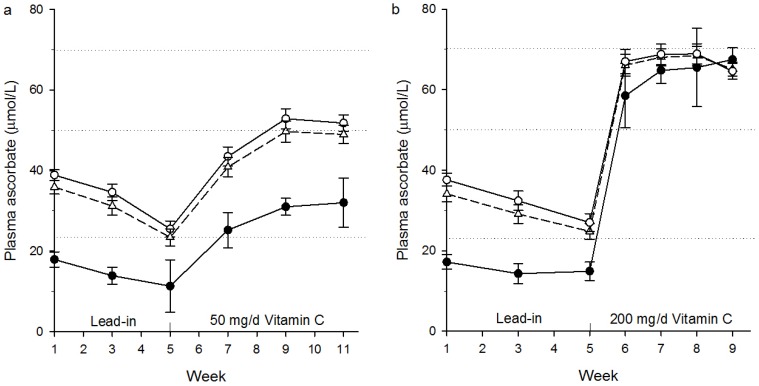
Plasma ascorbate concentrations in (**a**) the 50 mg/day vitamin C intervention group and (**b**) the 200 mg/day vitamin C intervention group. (**a**) Participants (Δ, *n* = 35) were supplemented with 50 mg/day vitamin C for six weeks; this group was subdivided into those with >23 μmol/L (○, *n* = 30) or ≤23 μmol/L (●, *n* = 5) plasma ascorbate at study entrance; (**b**) Participants (Δ, *n* = 35) were supplemented with 200 mg/day vitamin C for a minimum of four weeks; this group was subdivided into those with >23 μmol/L (○, *n* = 29) or ≤23 μmol/L (●, *n* = 6) plasma ascorbate at study entrance. Dotted lines demarcate ≤23 μmol/L ascorbate (hypovitaminosis C), ≥50 μmol/L ascorbate (‘adequate’), and ≥70 μmol/L ascorbate (saturating).

In the group supplemented with 50 mg/day vitamin C, the mean plasma ascorbate concentration for the >23 the μmol/L sub-group (*n* = 30) was 39 ± 7 μmol/L at entrance to the study. Following the lead-in phase the participants’ average plasma ascorbate concentration had dropped to 26 ± 10 μmol/L (baseline, Figure 1a). After six weeks’ supplementation with 50 mg/day vitamin C, this had risen to 52 ± 11 μmol/L, a level considered ‘adequate’ [13]. In contrast, the average plasma ascorbate concentration of the ≤23 μmol/L sub-group (*n* = 5), which was 18 ± 4 μmol/L at study entrance, reached only 32 ± 14 μmol/L following supplementation (Figure 1a). This is considered inadequate [3,13].

### 3.3. Plasma Ascorbate in the 200 mg/Day Intervention Group

In the group supplemented with 200 mg/day vitamin C, the mean plasma ascorbate concentration for the >23 μmol/L sub-group (*n* = 29) was 38 ± 9 μmol/L at entrance to the study. After the lead-in phase the participants’ average plasma ascorbate concentration had dropped to 27 ± 12 (baseline, Figure 1b). Within one week of supplementation at 200 mg/day vitamin C, their concentrations had risen to 67 ± 15 μmol/L and this was maintained for the four weeks of supplementation. The ≤23 μmol/L sub-group (*n* = 6), which was 17 ± 5 μmol/L at study entrance, reached plasma ascorbate concentrations of >60 μmol/L within two weeks of supplementation (Figure 1b).

Our data above indicate that, although individuals with ≤23 μmol/L plasma ascorbate do not reach ‘adequate’ levels with 50 mg/day vitamin C supplementation, they do respond to 200 mg/day supplementation, quickly reaching saturating or ‘optimal’ plasma levels.

### 3.4. Dietary Intakes of Vitamin C

Dietary intakes of vitamin C were determined for 80% of the participants through analysis of seven-day dietary records. No significant differences were observed between the vitamin C intakes for the >23 μmol/L and ≤23 μmol/L sub-groups at study entrance (*i.e*., ~30 mg/day vitamin C). Therefore, the difference in the plasma ascorbate status of these two sub-groups at study entrance cannot be explained by a difference in dietary intake (Table 2). Furthermore, although the participants had identical vitamin C consumption during the 50 mg/day intervention phase, there remained a significant difference in plasma ascorbate levels between the sub-groups. In contrast, no difference was observed in plasma ascorbate concentrations between the two sub-groups following 200 mg/day intervention. This suggests that factors other than dietary intake are contributing to the lack of response to 50 mg/day intervention in the individuals with hypovitaminosis C. When dietary vitamin C intake was assessed based on body weight, the ≤23 μmol/L sub-groups had an average intake of 0.3 ± 0.1 mg/kg/day while the >23 μmol/L sub-groups had an average intake of 0.4 ± 0.2 mg/kg/day at study entrance. These intakes were, however, not significantly different.

### 3.5. Factors Affecting Post Supplementation Ascorbate Concentrations

To investigate factors affecting the final plasma ascorbate concentration in the 50 mg/day supplementation group, backward stepwise multiple linear regression analysis was carried out. The participants’ initial plasma vitamin C concentration was a significant predictor of outcome, as was body weight (Table 3). BMI was excluded from the selection model, as weight and BMI were collinear. The regression model explained ~30% of the variability in the plasma ascorbate concentration following supplementation with 50 mg/day vitamin C.

## 4. Discussion

The data from our retrospective analysis indicate that individuals with hypovitaminosis C do not reach adequate ascorbate status (defined as ≥50 μmol/L [13]) upon supplementation with 50 mg/day vitamin C. Participants had comparable dietary vitamin C intakes at study entrance and following intervention; therefore, the attenuated plasma levels cannot be explained by differences in dietary intake. Our intervention was in addition to the baseline intake of the participants and it should be noted that, even with a total post-supplementation dietary intake of 75 mg/day, the individuals with hypovitaminosis C were not able to achieve adequate plasma ascorbate concentrations. In contrast, supplementation with the higher dose of 200 mg/day vitamin C overcame the attenuated response of the individuals with hypovitaminosis C.

International epidemiological studies have indicated a number of predictors of baseline vitamin C status, in addition to dietary intake; these include smoking status, gender, BMI or weight, and waist circumference or ratios [6,7,26,27,28,29,30,31,32,33,34,35,36]. Smokers have lower vitamin C status than non-smokers, even with comparable vitamin C intakes [37]. This is due to a faster metabolic turnover of ascorbate in smokers [38], likely due to increased oxidative stress, giving smokers a higher requirement (~35 mg/day) for dietary vitamin C [12]. However, all of our study participants were non-smokers, therefore, this does not explain the low response of some to supplementation.

Epidemiological studies consistently find that men have lower ascorbate status than women [7,26,27,28,29,32]. However, this is likely due to differences in body weight that result in a volumetric dilution effect [32]; a given dose in a larger volume produces a lower concentration, an effect which has been observed with increasing obesity [39]. Using stepwise multiple linear regression modelling we found that baseline ascorbate status and body weight accounted for about 30% of the post-supplementation variability. Similarly, Block *et al.* [40] found that prior ascorbate depletion and body weight affected ascorbate levels obtained following supplementation. Thus, individuals with higher body weight appear to have a higher requirement for vitamin C.

There are a number of limitations to our retrospective study, including the small size of the hypovitaminosis C sub-groups. Furthermore, the study cohort consisted primarily of young, lean males, and whether the findings can be generalized remains uncertain. This is because weight tends to increase with age, as do comorbidities, which affect the attainable and needed ascorbate concentrations. Thus, future studies would be strengthened by using larger numbers of hypovitaminosis C individuals supplemented in a prospective manner, and testing both genders and different age groups. Assessing relevant polymorphisms would also add strength to these studies.

Some of the unexplained variability in our participants’ response to supplemental vitamin C could be due to the effects of genetic variants on vitamin C uptake and/or metabolism. Circulating ascorbate status may be influenced by variants in the *SLC23A1* gene which encodes the sodium-dependent vitamin C transporter-1 (SVCT1) responsible for active uptake of ingested vitamin C through the intestinal epithelium [41]. A number of single nucleotide variants have been identified in the *SLC23A1* gene [42] and some of these result in a 40%–75% decrease in ascorbate transport in an *in vitro* oocyte model [43]. Although we did not test *SLC23A1* gene variants in our study participants, several variants have relatively common minor allele frequencies (0.25–0.32) in the Caucasian population [44]. These however, appear to have relatively minor effects on circulating ascorbate concentrations [44], and may only have a minimal impact on the poor response of hypovitaminosis C individuals to supplementation.

Ascorbate status could potentially be influenced by genetic variants affecting the metabolism of ascorbate [42]. For example, a variant of the haemoglobin-binding protein haptoglobin (Hp2-2) has a decreased ability to bind haemoglobin and results in increased oxidation of ascorbate *in vitro* [45]. Several studies have shown that this variant is associated with lower circulating ascorbate concentrations [45,46,47]. Since the Hp2-2 variant is fairly prevalent (0.33–0.39) in Caucasian populations [45,47], and appears to have a greater effect in individuals with dietary vitamin C intakes <90 mg/day [47], this variant may be a factor in the moderate response of our hypovitaminosis C participants who consumed <90 mg/day total vitamin C. Since high-dose vitamin supplementation has been shown to ameliorate certain gene variant defects [48], and is also hypothesized to occur with vitamin C-related variants [49], this could partly explain why the higher dose vitamin C intervention (200 mg/day) overcame the low response in the hypovitaminosis C participants. Therefore, individuals with genetic variants affecting ascorbate status may require higher dietary intakes. The prevalence and effects of genetic variants on ascorbate status and health need to be explored further.

Epidemiological studies indicate that 20% of the US population is at risk of developing vitamin C deficiency [6], and although this has not been determined within the Australasian population, the rates are likely to be similar. In support of this, our preliminary data from 389 middle aged men and women who were recruited randomly as part of the Canterbury Health, Ageing and Life Course (CHALICE) study [50], revealed that approximately 14% exhibited hypovitaminosis C (unpublished observations). The Australasian RDI is supposed to provide an intake of vitamin C sufficient to meet the requirements of 97.5% of healthy adults [11]. However, our data suggest that a significant proportion of the Australasian population may not reach adequate plasma vitamin C levels with the current RDI.

In their document on the nutrient reference values for Australia and New Zealand, the Australian Department of Health and Ageing and the New Zealand Ministry of Health have compiled Suggested Dietary Targets to reduce chronic disease risk [51]. This is a daily average intake from food and beverages for certain nutrients that may help in the prevention of chronic disease. It includes nutrients, such as vitamin C, for which there is a body of evidence supporting a potential “chronic disease preventative effect” at levels substantially higher than the EAR and RDI. The suggested dietary target for vitamin C is 220 mg for men and 190 mg for women [51]. This aligns well with our findings that 200 mg/day vitamin C provides saturating plasma concentrations of ascorbate [22] and optimal tissue status [23].

In our study we showed that supplementation of hypovitaminosis C individuals with 50 mg/day vitamin C, which provided a total vitamin C intake of 75 mg/day, was insufficient to provide adequate vitamin C status (*i.e*., ≥50 μmol/L) in these individuals. We also determined that increased body weight has a negative impact on ascorbate status. It has been previously suggested that vitamin C intakes be based upon body weight (*i.e*., mg vitamin C/kg body weight/day [40]). Although this would cater to the increasing trend in individualized health care, particularly in light of increasing obesity rates worldwide [52], it may be difficult to implement at a public health level. Nevertheless, the association between body weight and vitamin C status warrants further evaluation in an adequately powered prospective study.

## Figures and Tables

**Table 1:** Group characteristics at study entrance.

Measure	Total Cohort	50 mg/Day Group ^1^			200 mg/Day Group ^1^		
		50 mg/Day Group	>23 μmol/L Subgroup	≤23 μmol/L Subgroup	200 mg/Day Group	>23 μmol/L Subgroup	≤23 μmol/L Subgroup
Number	70	35	30	5	35	29	6
Ascorbate ^2^ (μmol/L)	35 ± 11	36 ± 10	39 ± 7	18 ± 4 **	34 ± 11	38 ± 9	17 ± 5 **
Age (years)	22 ± 4	22 ± 4	21 ± 4	22 ± 6	23 ± 4	22 ± 4	23 ± 5
Weight (kg)	84 ± 20	86 ± 22	84 ± 16	102 ± 40	81 ± 18	80 ± 14	85 ± 31
Height (cm)	180 ± 7	181 ± 7	181 ± 7	178 ± 10	180 ± 8	180 ± 7	177 ± 10
BMI (kg/m^2^)	26 ± 5	26 ± 6	26 ± 4	31 ± 9 *	25 ± 5	25 ± 4	27 ± 7

^1^ Vitamin C was derived from either vitamin C tablets or the equivalent dose from kiwifruit; ^2^ Participants with plasma ascorbate concentrations <50 μmol/L were recruited for the studies. Data represent mean ± SD; * *p* < 0.05 and ** *p* < 0.001 for unpaired *t*-test of ≤23 μmol/L sub-group compared with >23 μmol/L sub-group within each intake group.

**Table 2:** Vitamin C intake and ascorbate status for the 50 and 200 mg/day intervention groups at study entrance, baseline and post-intervention. These groups were subdivided into those with >23 μmol/L or ≤23 μmol/L plasma ascorbate at study entrance.

	Plasma Ascorbate (μmol/L) ^1^		Vitamin C Intake (mg/Day) ^1^	
	>23 μmol/L Sub-Group	≤23 μmol/L Sub-Group	>23 μmol/L Sub-Group	≤23 μmol/L Sub-Group
50 mg/day group	*n* = 30	*n* = 5	*n* = 30	*n* = 5
Entrance	39 ± 7	18 ± 4 **	33 ± 13	32 ± 16
Baseline	26 ± 10	11 ± 15 *	30 ± 14	25 ± 7
Intervention	52 ± 11	32 ± 14 **	75 ± 15	75 ± 10
200 mg/day group	*n* = 18	*n* = 4	*n* = 18	*n* = 4
Entrance	35 ± 7	17 ± 5 **	34 ± 19	28 ± 15
Baseline	25 ± 13	16 ± 6	28 ± 13	28 ± 10
Intervention	60 ± 8	67 ± 7	214 ± 18	230 ± 12

^1^ Data represent mean ± SD. Vitamin C was derived from either vitamin C tablets or the equivalent dose from kiwifruit; * *p* < 0.05 and ** *p* < 0.001 as determined by unpaired Student’s *t*-test of >23 μmol/L sub-group compared with ≤23 μmol/L sub-group.

**Table 3:** Backward stepwise multiple linear regression analysis to determine independent predictors of plasma ascorbate concentration in the 50 mg/day group.

Predictors	B (Non-Standardized) ^1^	β (Standardized) ^2^	*p*
Constant	56.4		0.00002
Initial plasma ascorbate (μmol/L)	0.426	0.325	0.033
Weight (kg)	−0.263	−0.430	0.006

^1^ The regression coefficient in the original measurement units; it indicates how much the dependent variable varies with a predictor when all other predictors are held constant; ^2^ The regression coefficient which refers to the number of standard deviation changes we would expect in the outcome variable for one standard deviation change in the predictor variable; it is a measure of how strongly each predictor variable influences the dependent variable. Plasma ascorbate concentration (μmol/L) post-intervention was the dependent variable. Only variables that remained in the final model are shown; variables that were entered into the stepwise regression but did not remain include age (y) and dietary intake (at baseline). The adjusted R squared value for the final model was 0.297.

## Abbreviations

The following abbreviations are used in this manuscript:

EAR		estimated average requirement
RDI		recommended dietary intake
